# Cytokine Response Patterns in Severe Pandemic 2009 H1N1 and Seasonal Influenza among Hospitalized Adults

**DOI:** 10.1371/journal.pone.0026050

**Published:** 2011-10-13

**Authors:** Nelson Lee, Chun Kwok Wong, Paul K. S. Chan, Martin C. W. Chan, Rity Y. K. Wong, Samantha W. M. Lun, Karry L. K. Ngai, Grace C. Y. Lui, Bonnie C. K. Wong, Sharon K. W. Lee, Kin Wing Choi, David S. C. Hui

**Affiliations:** 1 Department of Medicine and Therapeutics, Faculty of Medicine, The Chinese University of Hong Kong, Hong Kong, Special Administrative Region, People's Republic of China; 2 Stanley Ho Centre for Emerging Infectious Diseases, Faculty of Medicine, The Chinese University of Hong Kong, Hong Kong, Special Administrative Region, People's Republic of China; 3 Department of Chemical Pathology, Faculty of Medicine, The Chinese University of Hong Kong, Hong Kong, Special Administrative Region, People's Republic of China; 4 Department of Microbiology, Faculty of Medicine, The Chinese University of Hong Kong, Hong Kong, Special Administrative Region, People's Republic of China; Centre de Recherche Public de la Santé (CRP-Santé), Luxembourg

## Abstract

**Background:**

Studying cytokine/chemokine responses in severe influenza infections caused by different virus subtypes may improve understanding on pathogenesis.

**Methods:**

Adults hospitalized for laboratory-confirmed seasonal and pandemic 2009 A/H1N1 (pH1N1) influenza were studied. Plasma concentrations of 13 cytokines/chemokines were measured at presentation and then serially, using cytometric-bead-array with flow-cytometry and ELISA. PBMCs from influenza patients were studied for cytokine/chemokine expression using *ex-vivo* culture (Whole Blood Assay,±PHA/LPS stimulation). Clinical variables were prospectively recorded and analyzed.

**Results:**

63 pH1N1 and 53 seasonal influenza patients were studied. pH1N1 patients were younger (mean±S.D. 42.8±19.2 vs 70.5±16.7 years), and fewer had comorbidities. Respiratory/cardiovascular complications were common in both groups (71.4% vs 81.1%), although severe pneumonia with hypoxemia (54.0% vs 28.3%) and ICU admissions (25.4% vs 1.9%) were more frequent with pH1N1. Hyperactivation of the proinflammatory cytokines IL-6, CXCL8/IL-8, CCL2/MCP-1 and sTNFR-1 was found in pH1N1 pneumonia (2–15 times normal) and in complicated seasonal influenza, but not in milder pH1N1 infections. The adaptive-immunity (Th1/Th17)-related CXCL10/IP-10, CXCL9/MIG and IL-17A however, were markedly suppressed in severe pH1N1 pneumonia (2–27 times lower than seasonal influenza; *P*−values<0.01). This pattern was further confirmed with serial measurements. Hypercytokinemia tended to be sustained in pH1N1 pneumonia, associated with a slower viral clearance [PCR-negativity: day 3–4, 55% vs 85%; day 6–7, 67% vs 100%]. Elevated proinflammatory cytokines, particularly IL-6, predicted ICU admission (adjusted OR 12.6, 95%CI 2.6–61.5, per log_10_unit increase; *P* = 0.002), and correlated with fever, tachypnoea, deoxygenation, and length-of-stay (Spearman's rho, *P*-values<0.01) in influenza infections. PBMCs in seasonal influenza patients were activated and expressed cytokines *ex vivo* (e.g. IL-6, CXCL8/IL-8, CCL2/MCP-1, CXCL10/IP-10, CXCL9/MIG); their ‘responsiveness’ to stimuli was shown to change dynamically during the illness course.

**Conclusions:**

A hyperactivated proinflammatory, but suppressed adaptive-immunity (Th1/Th17)-related cytokine response pattern was found in severe pH1N1 pneumonia, different from seasonal influenza. Cytokine/immune-dysregulation may be important in its pathogenesis.

## Introduction

Seasonal influenza affects 5–10% of the world's population annually, causing 3–5 million severe infections and 250,000–500,000 deaths [1]. Typically, severe progressive influenza diseases occur in patients who are of advanced age and have pre-existing, immunocompromising conditions. In early 2009, a novel influenza A/H1N1 virus (pH1N1) emerged and rapidly caused a pandemic [1], [2], [3], [4]. It has been estimated that in some populations, up to 20–40% of individuals were affected, and had resulted in excessive hospitalizations and deaths [1], [2], [4]. In the United States, 195,000–403,000 persons were hospitalized for severe pH1N1 infection, and 8,870–18,300 died by April 2010 [1], [2], [4], [5]. The pH1N1 virus has continued to co-circulate with the seasonal influenza viruses in the community [1], [3]. While most patients develop mild upper respiratory-tract infection with pH1N1, some may progress to develop severe lower respiratory-tract complications such as pneumonia [1], [2], [4], [5], [6]. In contrast to seasonal influenza, young adults and previously healthy individuals may also develop severe diseases [2], [4], [5], [6]. Among hospitalized adults, about 9–34% may require intensive care because of progressive respiratory failure, which can be associated with high mortality (14–46%); notably, some of the manifestations (e.g. ARDS, hemophagocytosis) are quite similar to those of H5N1 influenza [2], [4], [5], [6], [7], [8], [9].

Despite its burden, the pathogenesis of severe influenza disease remains poorly understood and the management approach uncertain. In particular, progressive pneumonitis caused by pH1N1 is somewhat difficult to explain since the virus is neither shown to have markedly increased virulence over seasonal influenza viruses, nor it is a particularly strong cytokine inducer *in vitro* [2], [4], [10], [11], [12], [13]. Earlier reports indicated the presence of an exuberant inflammatory cytokine response in severe pH1N1 pneumonia [8], [9], [10], [11], [13]; however how these responses compared with seasonal influenza is unknown. In this study, we examined the cytokine response patterns in adults hospitalized for severe infections caused by pH1N1 and seasonal influenza viruses. Their relations to disease severity and outcomes were studied. Such information may provide important insights into the immunopathogenic mechanisms of severe influenza disease, which may lead to improved patient management in future.

## Methods

### Patients and sampling

We prospectively studied adults aged ≥17 years who were hospitalized for laboratory-confirmed influenza infection during year 2008 (two seasonal peaks)[3], [7] and 2009 (first wave of pH1N1 influenza)[3], [8]. Hospital admission procedures for both seasonal and pH1N1 influenza patients have been described [7], [8]. In brief, patients presenting with acute febrile respiratory illnesses would be considered for hospitalization if they had developed potentially serious medical conditions/complications, exacerbation of underlying illnesses, or severe constitutional and respiratory symptoms that were unmanageable at home. In all hospitalized patients, nasopharyngeal-aspirates were collected to test for influenza virus, regardless of perceived etiology. For seasonal influenza, the diagnosis was established by immunofluorescence assay [7]; for pH1N1 influenza, the diagnosis was established by a specific RT-PCR assay [8]; virus isolation was performed in parallel in all cases for confirmation (see below). Once diagnosed (seasonal influenza A or B, or pH1N1), patients were identified and enrolled by the research team on a daily basis [7], [8].

After obtaining informed written consent, peripheral venous blood samples were taken immediately at the time of recruitment (before antiviral therapy, if given), and then twice during the period of hospitalization until the end of first week or discharge. Blood samples were subjected to cytokine/chemokine assays, inflammatory-marker assays, and *ex vivo* PBMC cytokine/chemokine production studies (see below). Nasal/pharyngeal swabs were also taken for influenza viral RNA detection at the same time points. Clinical information and outcomes were recorded systemically, using a standardized research tool identical for both the seasonal and pH1N1 influenza cohorts [7]. Ethical approval for study was obtained from the Institutional Review Boards of the Chinese University of Hong Kong and the Hospital Authority of Hong Kong.

### Virological investigations

All nasopharyngeal-aspirates collected at presentation were subjected to an immunofluorescence assay for influenza A and B, RSV and parainfluenza viruses detection as described Directigen™ Flu A+[7], [8], [14], [15]. In 2009, a real-time RT-PCR assay specific for the haemagglutinin gene of the pH1N1 virus was additionally performed for diagnosis, using established method [8]. In all cases, virus isolation was performed in parallel using Madin-Darby canine kidney cells. Subsequent subtyping of virus isolates (seasonal H1N1, H3N2; pH1N1) was performed by the National Influenza Centre at the Centre for Health Protection in Hong Kong [3], [7], [8]. Viral RNA detection in the serially collected nasal/pharyngeal swabs was performed using real-time RT-PCR assays (which targeted the M-gene of influenza viruses) as previously described [8], [14], [15].

### Assays of plasma concentrations of cytokines/chemokines and inflammatory-markers

EDTA blood samples were immersed in ice and transported immediately to a biosafety level-II laboratory for processing. Plasma was separated by centrifugation (2000 *g* for 10 min) at 4°C and stored in 300 µL aliquots at −70°C until analysis. Plasma concentrations of 10 cytokines/chemokines including interleukin(IL)-1β, IL-6, IL-10, IL-12p70, tumour necrosis factor(TNF)-α, CXCL8/IL-8, CXCL9/MIG (monokine induced by interferon-γ), CXCL10/IP-10 (interferon gamma-induced protein-10), CCL2/MCP-1 (monocyte chemoattractant protein-1), and CCL5/RANTES, were assayed using cytometric bead array (CBA) reagents (BD Pharmingen, San Diego,CA) with four-color FACSCalibur flow-cytometer (BD Biosciences Corp, San Jose,CA) as previously described [8], [15]. In CBA, different bead populations with distinct fluorescence intensities had been coated with capturing antibodies specific for different cytokines/chemokines. After incubation with 50 µL plasma, the cytokine/chemokine captured beads were mixed with phycoerythrin-conjugated detection antibodies to form sandwich immune-complexes. Fluorescence flow-cytometry of the beads provides simultaneous quantification of a panel of cytokines/chemokines. In addition, plasma concentrations of interferon(IFN)-γ, sTNFR1 (soluble tumour necrosis factor receptor 1, which indirectly indicates TNF-α release) and interleukin(IL)-17A were quantified by ELISA (R&D Systems Inc., MN,USA)[8], [15]. These cytokines/chemokines were selected for study based on our previous research and recent literature findings [2], [8], [9], [10], [11], [13]. The cytokine/chemokine assays used were identical for both seasonal and pH1N1 influenza cohorts. Normal plasma reference ranges of cytokines/chemokines (listed in **Table 1**) were established from >100 healthy adults [15].

**Table 1:** Plasma cytokine/chemokine concentrations in adults hospitalized for seasonal or pandemic H1N1 influenza, measured at presentation.

Cytokine or chemokine [plasma reference range, pg/mL]	Seasonal Flu A (n = 45)^1^	Seasonal Flu A/H3N2 (n = 35)^2^	Seasonal Flu A/H1N1 (n = 10)	Seasonal Flu B (n = 8)	Pandemic Flu A/H1N1, severe (n = 34)^3^	Pandemic Flu A/H1N1, mild (n = 29)^4^
IL-6 [<3.1]	14.6 (6.2–40.2)	14.3 (6.3–38.9)	20.4 (4.7–93.3)	12.5 (6.6–19.6)	45.8 (7.8–167.6)**	2.7 (1.4–4.7)**
CXCL8/IL-8 [<5.0]	19.7 (9.9–53.8)	32.1 (10.3–63.0)*	12.8 (7.7–27.0)	17.6 (5.0–44.7)	20.1 (11.6–39.4)	7.8 (5.0–11.0)**
CCL2/MCP-1 [<10.0–57.0]	121.3 (84.4–176.5)	121.3 (89.2–167.0)	105.5 (76.2–205.7)	148.8 (64.8–305.4)	102.7 (52.9–315.2)	24.0 (11.3–38.3)**
sTNFR-1 [484–1407]	2237.1 (1414.8–3356.8)#	2399.6 (1612.4–3285.4)*	1610.1 (1099.4–2438.7)	1316.5 (1198.9–1737.9)	2210.8 (1250.7–3390.6)	1048.1 (831.1–1376.4)**
IL-10 [<7.8]	4.0 (2.1–6.2)	4.4 (2.9–6.9)*	1.8 (0.5–3.8)	3.5 (1.8–21.1)	6.1 (3.4–12.0)*	1.9 (1.2–2.5)**
CXCL10/IP-10 [202–1480]	14419 (6984–39756)**	26154 (7891–44464)#	9042 (4625–16012)	4255 (1476–8769)	1678 (835–2835)**	3614 (782–6538)
CXCL9/MIG [48.0–482.0]	6609.0 (475.5–21092.6)**	12154.3 (576.8–26173.6)*	2163.2 (182.8–6905.6)	171.5 (103.8–380.6)	247.5 (118.7–773.2)**	258.4 (179.2–1386.4)
IL-17A [<10.0]	29.1 (16.3–43.5)**	27.8 (18.5–42.7)	31.7 (9.3–63.0)	53.3 (50.2–72.9)	15.4 (5.0–17.5)**	17.1 (7.5–19.5)
CCL5/RANTES [4382–18783]	4580.8 (1730.4–7710.3)	4917.7 (1690.1–7903.9)	2988.6 (1599.8–7066.8)	6212.4 (2386.7–9084.6)	2780.8 (1645.3–4587.3)#	3341.2 (1923.6–4758.0)
IFN-gamma [<15.6]	0.5 (0.5–10.8)*	0.5 (0.5–10.9)	0.5 (0.5–12.1)	18.9 (6.1–30.5)	0.5 (0.5–0.5)	0.5 (0.5–0.5)
TNF-alpha [<10.0]	1.7 (1.2–2.3)	1.5 (1.1–2.6)	1.8 (1.7–2.2)	1.7 (1.4–2.7)	0.5 (0.5–1.4)	0.5 (0.5–1.3)
IL-12 [<7.8]	1.0 (0.5–1.7)	0.5 (0.5–1.6)	1.5 (1.0–2.5)	1.5 (0.7–3.5)	0.5 (0.5–0.5)	0.5 (0.5–1.3)
IL-1 beta [<3.9]	2.2 (1.9–2.8)	2.2 (1.8–2.8)	2.6 (2.1–2.9)	2.2 (1.5–5.0)	0.5 (0.5–2.4)	0.5 (0.5–0.5)
Acute phase reactants						
C-reactive protein [<9.9 mg/L]	42.6 (13.5–87.2)	30.7 (12.4–96.9)	52.5 (19.4–187.3)	30.1 (14.9–55.3)	53.6 (28.4–172.0)	11.0 (3.8–13.1)
Serum amyloid A [1000–5000 ng/mL]	10794.3 (10308.7–11262.9)	10829.2 (10292.8–11197.5)	10757.6 (10259.4–11548.7)	11493.5 (10977.9–11809.0)	--	--
Cortisol [23.2–118.5 ng/mL]	435.0 (253.0–555.0)	435.0 (270.0–597.0)	405.0 (243.3–595.8)	362.0 (344.5–532.5)	--	--

Values are stated as median (interquartile range, IQR); pandemic influenza A/H1N1 ‘severe’: radiographic pneumonia plus hypoxemia; ‘mild’: hospitalized for significant respiratory or systemic symptoms (only 5/29 patients had mild pulmonary infiltrates on chest radiographs)[8]. Over 80% of seasonal influenza patients had respiratory/cardiovascular complications, and nearly half developed hypoxemia. Only 2 patients received long-term immunosuppressants in these cohorts. The normal plasma reference ranges of cytokines/chemokines were obtained from >100 healthy individuals [8], [15].

Comparisons: (**1**) Seasonal influenza A (combined) vs influenza B; (**2**) seasonal influenza A/H3N2 vs A/H1N1; (**3**) severe pH1N1 vs all seasonal influenza cases (similar results when influenza B was excluded); (**4**) pH1N1 cases, ‘severe’ vs ‘mild’. Fewer pH1N1 infections had detectable levels of IFN-γ compared with seasonal influenza (8.8% vs 43.4%; p<0.001). Cytokine/chemokine concentrations were also compared between severe pH1N1 pneumonia and a subgroup of seasonal influenza patients with complicated infections and hypoxemia: CXCL10/IP-10, CXCL9/MIG and IL-17A concentrations were all significantly lower in severe pH1N1 infections (all p<0.01), and fewer had detectable IFN-γ level (p = 0.001). Complete data on plasma C-reactive protein (n = 34), serum amyloid A and cortisol were unavailable for pH1N1 cases. Mann-Whitney U test,

**p≤0.01,

*p<0.05;

#p<0.10.

Inflammatory-markers (‘acute-phase reactants’) and cortisol levels were also studied. Serum amyloid A and cortisol were quantified by ELISA (Antigenix America Inc, NY,USA and ENZO Life Sciences Int’l, PA,USA). Plasma C-reactive protein was measured using enzymatic colorimetric assay (Roche-Hitachi D–P Modular Analyzer, Roche Diagnostics GmbH, Mannheim,Germany).

### *Ex vivo* production of cytokines/chemokines using Whole Blood Assay (WBA)

To evaluate the role of inflammatory cells in mediating the cytokine/chemokine responses, the Whole Blood Assay (WBA) method was used to study cytokine production *ex vivo* by the peripheral blood mononuclear cells (PBMC) collected from patients during the course of seasonal influenza infection (similar studies for pH1N1 infection have been planned). WBA has been used to study cytokine responses mediated by the inflammatory cells in patients with other naturally-occurring infections [16], [17], [18]. It provides a natural milieu to study cytokine production, preserving the intercellular interactions and the circulating stimulatory/inhibitory mediators (including the soluble receptors), at their physiological concentrations [16]. Briefly, within 1 hour of EDTA blood collection (kept at room temperature), blood was diluted 1∶1 with RPMI 1640 (Gibco Laboratories, NY,USA), and 0.5 ml aliquots were deposited in each well of a 24-well plate (Nalge Nunc International, IL,USA). The blood culture was then incubated *with or without* phytohaemagglutinin (PHA)(Sigma, MO,USA), at 10 µg/ml; and lipopolysaccharide (LPS), at 25 µg/ml (Sigma), for 24 h at 37°C in a 5% CO2 atmosphere. After incubation, the cell-free supernatant was harvested and stored at −70°C until CBA for cytokines/chemokines. PHA is a specific T cell mitogen, and LPS is a B cell and macrophage mitogen, and a toll-like receptor 4 ligand. Concentrations of 12 cytokines/chemokines released *ex vivo* by the PBMC with or without PHA/LPS stimulation were measured and reported. The fold-change in increased expression after stimulation (i.e., the ‘responsiveness’) was calculated for each cytokine/chemokine (post-stimulation concentration÷ baseline concentration)[16].

### Statistical analysis

Plasma concentrations of cytokines/chemokines measured at presentation were described (median and interquartile range, IQR) and compared between infections caused by different influenza virus subtypes using the *Mann-Whitney U* test [15]. Patients with severe pH1N1 pneumonia (defined as radiographic pneumonia and hypoxemia, with requirement of supplemental oxygen therapy to maintain oxygen saturation >95%) were also compared to those with milder illness [7], [8]. Correlations between plasma cytokine/chemokine concentration and clinical variables were analyzed using the Spearman's rank correlation coefficient *rho* (ρ)[8], [15]. To determine independent factors associated with intensive care unit (ICU) admission, variables with P-values ≤0.1 in univariate analyses were entered into logistic regression models; adjusted odds ratios (OR) and 95% confidence intervals (CI) were calculated and reported for explanatory variables[8], [14], [15]. *Ex vivo* PBMC cytokine production with or without PHA/LPS stimulation were compared using the *Mann-Whitney U* test [16]. The trend change in cytokine expression by PBMC across the study time points was analyzed using the *Jonckheere-Terpstra* test. In all analyses, a P-value of<0.05 was considered to indicate statistical significance. All probabilities were 2-tailed. Statistical analysis was performed using the PASW Statistics software, version 17.0 (SPSS Inc., Chicago,USA).

## Results

### Description of study cohorts

Altogether 63 pH1N1 and 53 seasonal influenza (A = 45, B = 8) patients were studied. Patients with pH1N1 influenza were younger (mean±S.D. 42.8±19.2 vs 70.5±16.7 years; *P*<0.001), and less frequently had underlying medical conditions (30% vs 53%; *P* = 0.013). Gender distribution was not different (male, 50% vs 57%). Both cohorts were hospitalized for severe, complicated influenza diseases: respiratory/cardiovascular complications 71.4% vs 81.1%, p = 0.224; oxygen desaturation 55.6% vs 49.1%, p = 0.485; radiographic evidence of pneumonia 57.1% vs 41.5%, p = 0.093. ‘Severe pneumonia’, as defined by radiographic pneumonia plus hypoxemia, was more common with pH1N1 influenza (54.0% vs 28.3%; *P* = 0.009). The ICU admission rate was also significantly higher (25.4% vs 1.9%; *P*<0.001). Two (3.2%) pH1N1 patients died during the course of hospitalization. There was no death in the seasonal influenza cohort.

### *In vivo* cytokine/chemokine responses in seasonal and pandemic H1N1 influenza

Plasma cytokine/chemokine concentrations measured at presentation for the pH1N1 and seasonal influenza groups are shown in **Table 1**. In patients with severe pH1N1 pneumonia, the proinflammatory cytokine IL-6 was highly elevated [about 15 times above its reference range (up to 54 times) for normal subjects [8], [15]; and 3–4 times higher than the seasonal influenza group, *P*<0.05]; there were also increases in CXCL8/IL-8, CCL2/MCP-1, and sTNFR-1 [about 2–4 times above their normal reference ranges (up to 8 times)]. Notably, when compared with the seasonal influenza group, the Th1-related CXCL10/IP-10 and CXCL9/MIG, and the Th17-related IL-17A were markedly suppressed in cases of severe pH1N1 pneumonia (2–27 times lower; *P*−values <0.01). Similar results were obtained when only complicated infections with hypoxemia were compared (**Table 1** **and footnotes**). The measured CXCL9/MIG values were mostly within the normal reference range, although CXCL10/IP-10 and IL-17A generally showed some increases. In addition, IFN-γ was less frequently detected in the severe pH1N1 pneumonia group (*P*<0.001); but a relative increase in IL-10 was observed (*P*<0.05). In mild pH1N1 infections without pneumonia, hypercytokinemia was largely absent; most measured values did not exceed the normal reference ranges.

When compared between different seasonal influenza virus infections (**Table 1**), it was found that plasma levels of CXCL9/MIG, CXCL8/IL-8, sTNFR-1 and CXCL10/IP-10 were higher in the influenza A/H3N2 subgroup than in the seasonal influenza A/H1N1 subgroup (by 2–4 times). Greater IL–17A and interferon-γ responses were observed in influenza B than influenza A patients, but the CXCL9/MIG, CXCL10/IP-10 and sTNFR-1 levels were lower (by 2–30 times).

**Figure 1:**
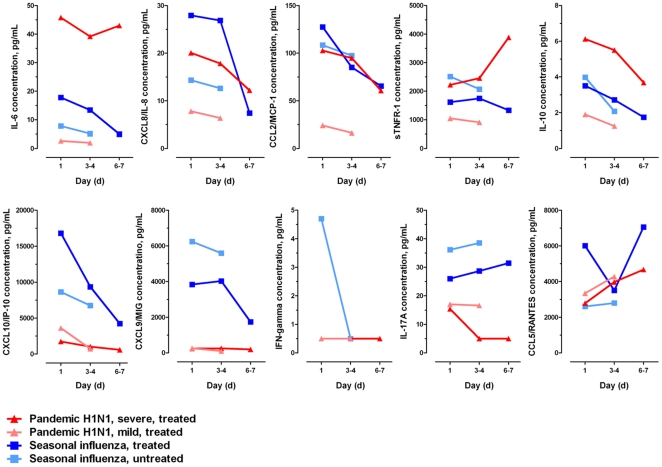
Serial changes in plasma cytokine/chemokine concentrations during the course of hospitalization. There was sustained elevation of the proinflammatory cytokines (IL-6, CXCL8/IL-8, CCL2/MCP-1, sTNFR-1) in severe pH1N1 pneumonia; the adaptive-immunity related cytokines (CXCL10/IP-10, CXCL9/MIG, IL-17A) were markedly suppressed compared with seasonal influenza. All patients with pH1N1 influenza (severe pneumonia, n = 34; milder illness, n = 29) received antiviral treatment soon after hospitalization/recruitment; none had received high-dose corticosteroids or other immunosuppressants for ‘viral pneumonitis’ or ‘ARDS’[8]. Among seasonal influenza patients (most had complicated illnesses, see Table 1 footnotes), 30(57%) received antiviral treatment. Median concentrations at each time point are shown for each group; the interquartile ranges (presented in Table 1) are omitted here for clarity. Fewer mild pH1N1 and untreated seasonal influenza patients remained hospitalized at day 6–7 for study (Day 1, n = 116; Day 3–4, n = 62; Day 6–7, n = 30).

Serial changes in the plasma cytokine/chemokine concentrations in patients with pH1N1 and seasonal influenza infection during their first week of hospitalization are described in **Figure 1**. There was sustained activation of the proinflammatory cytokines (IL-6, CXCL8/IL-8, CCL2/MCP-1, sTNFR-1) in severe pH1N1 pneumonia; the CXCL10/IP-10, CXCL9/MIG and IL-17A responses remained largely suppressed during the hospital course. Cytokine activation remained absent with mild pH1N1 infection. In seasonal influenza, plasma cytokine/chemokine levels (e.g. IL-6, CXCL-8/IL-8, CXCL10/IP-10, CXCL9/MIG) tended to decline rapidly after hospitalization, especially among the antiviral-treated patients. Increased CCL5/RANTES level was noted during the recovery phase of influenza infections. Sustained hypercytokinemia in pH1N1 influenza was noted to correspond to a slower viral clearance in the respiratory tract compared with seasonal influenza: PCR-negativity in nasal/pharyngeal swabs was found in 55% and 85% on day 3–4, and 67% and 100% on day 6–7 respectively.

There was evidence of collective activation of the pro-inflammatory cytokines/chemokines in both pH1N1 and seasonal influenza: plasma IL-6 concentration correlated significantly with that of CXCL8/IL-8 (Spearman's *rho* ρ+0.623, *P*<0.001), CCL2/MCP-1 (ρ+0.588, *P*<0.001), and sTNFR-1 (ρ+0.538, *P*<0.001). The Th1-related chemokines CXCL10/IP-10 and CXCL9/MIG were also closely correlated (ρ+0.823, p<0.001)(**Supplementary Material S1**). There was, however, little correlation between IL-6 and CXCL10/IP-10, CXCL9/MIG, or IL–17A levels. C-reactive protein, serum amyloid A and cortisol levels were all found to be elevated during influenza infection (**Table1**). Plasma C-reactive protein level significantly correlated with IL-6 (ρ+0.609, *P*<0.001) and sTNFR-1 (ρ+0.462, *P*<0.001) levels, but not with other cytokines/chemokines.

### Clinical correlations of the cytokine responses

For pH1N1 influenza, high plasma IL-6 concentration at presentation (adjusted OR 12.6, 95%CI 2.6–61.5, per log10 unit increase; *P* = 0.002) was independently associated with ICU admission, adjusted for age, comorbidity and time interval from onset-to-presentation. Similar associations with ICU admission were found for CXCL8/IL-8 and CCL2/MCP-1. Circulating IL-6, CXCL8/IL-8, CCL2/MCP-1, and sTNFR-1 levels were also found to correlate significantly with fever, tachypnoea, oxygen desaturation and hospital length-of-stay (**Table 2**).

**Table 2:** Correlations between initial cytokine/chemokine concentrations and clinical parameters at presentation (temperature, respiratory rate, oxygen saturation) and the clinical outcomes (hospital length-of-stay, ICU admission).

	Temperature (°C)	Respiratory Rate (breaths per min)	Oxygen saturation (SaO_2_%)	Length-of-stay (LOS, days)	Risk of ICU admission (adjusted OR, 95%CI)
Pandemic H1N1 influenza					
IL-6	+0.504**	+0.607**	−0.391**	+0.621**	12.6, 2.6–61.5**
CXCL8/IL-8	+0.480**	+0.420**	−0.506**	+0.528**	17.0, 1.9–151.9**
CCL2/MCP-1	+0.358**	+0.442**	−0.513**	+0.559**	60.9, 3.7–1000.0**
sTNFR-1	+0.368**	+0.511**	−0.473**	+0.424**	1.3, 0.5–3.2
IL-10	+0.447**	+0.632**	−0.513**	+0.548**	23.6, 2.5–226.9**
CXCL10/IP-10	+0.262*	–0.155	+0.020	–0.143	1.2, 0.4–3.9
CXCL9/MIG	+0.111	–0.154	–0.108	–0.014	1.0, 0.3–3.3
IL-17A	+0.135	–0.081	–0.167	–0.059	0.8, 0.2–3.5
CCL5/RANTES	–0.072	–0.030	–0.157	0.000	1.1, 0.1–13.3
					
Seasonal influenza					
IL-6	+0.351**	--	–0.342*	+0.371**	--
CXCL8/IL-8	+0.110	--	–0.202	+0.410**	--
CCL2/MCP-1	+0.179	--	–0.084	+0.064	--
sTNFR-1	+0.086	--	–0.114	+0.466**	--
IL-10	+0.002	--	–0.108	+0.044	--
CXCL10/IP-10	+0.076	--	–0.337*	+0.389**	--
CXCL9/MIG	–0.125	--	–0.182	+0.265	--
IL-17A	+0.178	--	+0.231	–0.229	--
CCL5/RANTES	+0.059	--	–0.052	–0.231	--
C-reactive protein	+0.104	--	–0.411*	+0.474**	--

For correlations with temperature, respiratory rate, oxygen saturation, and length-of-stay, the Spearman's rank coefficients (*rho*) were shown. For risk of ICU admission, the adjusted odds ratio and the 95% confidence interval (CI) per log_10_ unit increase in cytokine concentration were shown (adjusted for age, comorbidity and time from onset). Data on respiratory rate was incomplete in seasonal influenza cases, and there were too few ICU admissions to allow meaningful analysis.

*p<0.05,

**p<0.01.

For seasonal influenza, significantly higher plasma IL-6, CXCL8/IL-8, sTNFR-1, CXCL10/IP-10 and C-reactive protein concentrations were found in patients with respiratory/cardiovascular complications (all *P*−values<0.05). Plasma cytokine levels, particularly for IL-6, were found to correlate significantly with fever, oxygen desaturation and length-of-stay (**Table 2**). There were trends to suggest lower plasma IL–17A concentration being associated with respiratory/cardiovascular complications [median(IQR) 30.0(16.7–50.2) vs 43.4(27.7–62.3) pg/ml], oxygen desaturation (ρ+0.231) and longer length-of-stay (ρ−0.229), but these did not reach statistical significance.

### PBMC activation and cytokine/chemokine production in naturally-occurring influenza

**Figure 2:**
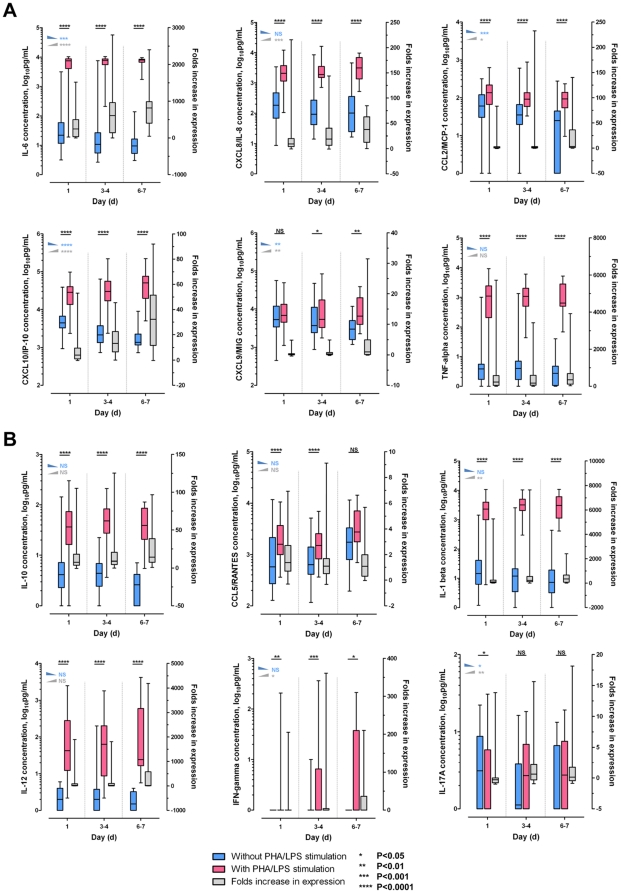
PBMC activation and *ex vivo* cytokine/chemokine expression during seasonal influenza infection. PBMC actively expressed IL-6, (CXCL8/IL-8), CCL2/MCP-1, CXCL10/IP-10, and CXCL9/MIG during acute influenza infection; upon illness recovery, cytokine production decreased, and there was a corresponding increase in cellular responsiveness to stimuli. Cytokine response pre-/post-stimulation and the trend changes in cytokine expression across time points (with PHA/LPS stimulation – red bars; without stimulation – blue bars; folds increase in expression or the ‘responsiveness’ – gray bars) were compared using the *Mann-Whitney U* test (asterisks, underlined), and the *Jonckheere-Terpstra* test (blue/gray triangles and asterisks), respectively. IL-17A did not appear to be activated via the PHA/LPS stimulation pathway.

We studied *ex vivo* cytokine/chemokine production of PBMC in seasonal influenza infection using WBA (n = 53). There were significant correlations found between *ex vivo* cytokine production and the *in vivo* plasma concentrations measured for IL-6 (Spearman's *rho* ρ+0.329, *P* = 0.017), CCL2/MCP-1 (ρ+0.568, *P*<0.001), CXCL10/IP-10 (ρ+0.597, *P*<0.001), CXCL9/MIG (ρ+0.784, *P*<0.001), and CCL5/RANTES (ρ+0.580, *P*<0.001). In the absence of PHA/LPS stimulation, *ex vivo* IL-6, CXCL8/IL-8, CCL2/MCP-1, CXCL10/IP-10 and CXCL9/MIG productions were found to be highest at baseline, and lowered in the serial samples obtained during recovery. With PHA/LPS stimulation, there was greatly increased secretion of these cytokines from the PBMC; and their ‘responsiveness’ (see Methods) was shown to restore/increase during the recovery phase (**Figure2a and 2b**).

To supplement these findings, we report evidence of activation and enhanced expression of the signaling molecule phospho-p38 mitogen activated protein kinase (MAPK) in the blood mononuclear cells during naturally-occurring pH1N1 infection in addition to seasonal influenza infection; p38 MAPK has been implicated in the signaling process of the cytokine response in influenza [15]. Details of the results are provided in **Supplementary Material S1**.

## Discussion

Our study reported different cytokine response patterns in adults hospitalized for severe pandemic H1N1 and seasonal influenza. In patients with pH1N1 pneumonia, there was sustained hyperactivation of the innate, proinflammatory cytokines (e.g. IL-6, CXCL8/IL-8, CCL2/MCP-1, sTNFR-1), which correlated with disease severity and outcomes; and in comparison with seasonal influenza, the adaptive Th1/Th17-immunity related cytokines (e.g. CXCL10/IP-10, CXCL9/MIG, IL-17A) were markedly suppressed. These findings suggested that cytokine dysregulation may play a role in disease pathogenesis.

Our results agree with recent *in vitro* and *in vivo* studies which have shown that an exuberant proinflammatory, innate cytokine response is the hallmark of severe influenza disease, in both seasonal and pandemic viruses infections [8], [9], [13], [19], [20], [21], [22], [23], [24], [25]. IL-6, one of the key proinflammatory cytokines, mediates fever, acute-phase reactions, and in experimental models shown to induce excessive tissue inflammation and dysregulate the immune response towards influenza virus [8], [9], [13], [19], [20], [25], [26], [27]. TNF-α (indicated by circulating sTNFR-1) mediates severe systemic symptoms, and aggravates lung tissue destruction[13], [19], [25]. CCL2/MCP-1 and CXCL8/IL-8 have been shown to recruit and activate monocytes/macrophages [8], [9], [19], [24], [25] and neutrophils [15], [19], [24] respectively in models of influenza pneumonia, leading to progression to ARDS. Our study adds that these cytokines/chemokines correlated significantly with severity of symptoms including high fever, tachypnoea and oxygen desaturation, and associated with adverse clinical outcomes such as ICU admission and longer duration of hospitalization (**Table 2**)[8], [9], [15], [19], [20], [21], [26], which indicated their possible pathogenic roles. However, while in seasonal influenza these proinflammatory responses are soon downregulated, they tend to be more sustained in cases of pH1N1 pneumonia [8], [21], [25], [28](**Figure 1**). We have previously shown that active viral replication may lead to continuous cytokine activation [8], [15]; and it is likely that the high viral burden and the more prolonged viral replication in pH1N1 pneumonia have driven a sustained cytokine response, resulting in progressive tissue inflammation [2], [4], [8], [9], [13], [15], [21], [29]. These findings on the host response do not contradict with the *in vitro* studies which have shown that intrinsically, the pH1N1 virus is a weaker cytokine inducer than H5N1, but more comparable to the seasonal influenza viruses [11], [12], [28], [30]. In fact, we observed little difference between the initial levels of most of the proinflammatory cytokines (except for IL-6, see Results) in severe pH1N1 and complicated seasonal infections [11]; and the adaptive-immunity related cytokines were lower in pH1N1 (see below). Although sample sizes were small in the subgroups, we have shown that the cytokine/chemokine responses in naturally-occurring seasonal influenza caused by different virus subtypes are generally consistent with those reported in *in vitro* studies, and in line with their known symptom severity (influenza A>B; H3N2>H1N1)[11], [22], [23], [24], [30].

Our finding of a strong adaptive-immunity related cytokine response (e.g. CXCL10/IP-10, CXCL9/MIG, IL–17A; and IFN-γ) in seasonal influenza but a relatively suppressed response in severe pH1N1 pneumonia is striking. CXCL10/IP-10 and CXCL9/MIG are stimulated by IFN-γ, which together orchestrate the Th1 cellular immune response, known to be important for virus control in influenza infection [21], [31]. IL-17A is a key cytokine involved in the newly described Th17 adaptive cellular immunity; its role in host defense against pathogens including influenza virus is increasingly recognized [9], [20], [32], [33], [34], [35], [36]. Bermejo-Martin JF et al and To KK et al have reported high levels of proinflammatory cytokines (e.g. IL-6, CXCL8/IL-8, CCL2/MCP-1) in critically ill pH1N1 patients; but IL–17A only showed a small increase, and in fact it was higher in the milder cases, correlating with enhanced viral clearance [9], [20]. Insignificant rise in IL–17A and IFN-γ was reported by others [19], [26]. Although CXCL10/IP-10 generally showed an increase [9], [19], [20], our results showed that the response was much lower than that in seasonal influenza. Recent studies have shown that numerous genes responsible for adaptive-immunity and T-cell response are downregulated in severe pH1N1 pneumonia [21], [26]; the T-cell functions are impaired, and some subsets including the Th17 cells are lost [31], [32], [37]. The impaired adaptive cellular immunity might have contributed to the delay in viral clearance in pH1N1 pneumonia, which in turn leads to sustained activation of the proinflammatory response [8], [13], [15], [20], [30], [31], [38]. Some of the inflammatory/anti-inflammatory cytokines released (e.g. IL-6, IL-10) might further dysregulate or inhibit the adaptive immunity, thus forming a vicious cycle [9], [13], [20], [21], [27]. Underlying immunocompromising conditions of the host might worsen these processes [14], [15]. A similar hypothesis has been put forward for H5N1 disease [11], [13], [29], [39]. Pathogenesis of severe influenza is complex, involving interactions between different viral (e.g. genetic constellation, virulence, immune evasion, tropism) and host factors (e.g. innate and adaptive responses, prior or cross-immunity, immunosuppression); but our data suggested that cytokine/immune dysregulation likely plays a role. Further studies are warranted [11], [13], [23], [24], [27], [30], [38], [39].

*Ex vivo* cytokine production study was performed to investigate the role of the immune cells in mediating the cytokine response during seasonal influenza infection. We found significant correlations between the cytokines/chemokines produced by the mononuclear cells and the plasma assay results, and there were dynamic changes of cytokine expression and cellular responsiveness throughout the course of illness (**Figure 2**). These findings suggested that the immune cells are activated, and may be responsible for part of the cytokine response observed during naturally-occurring infections. Macrophage/monocyte (in addition to pneumocytes) have been shown in experimental models as a key mediator of the proinflammatory responses, and lymphocytes (particularly the T-cells) being crucial in the adaptive, cellular immunity against influenza [13], [21], [25], [30], [31], [36], [37]. Similar studies on pH1N1 infection have been planned; but available data suggested that the results might be different (e.g. T-cell anergy to PHA stimulus), which clearly deserves further investigation [31], [32], [37].

Our findings may have important implications. Since the adaptive, cellular immunity and the associated cytokine responses are impaired/downregulated in pH1N1 pneumonia, further immunosuppression (e.g. with high-dose corticosteroid), is unlikely beneficial and may even be harmful. This is supported by recent clinical studies which have shown that corticosteroid use may lead to delayed viral clearance, pneumonia progression, bacterial superinfection and adverse outcomes in influenza infections [14], [40], [41], [42]. As such, future research on adjunctive therapy for severe influenza should consider targeting the proinflammatory pathways, while preserving immunity for virus control [13], [30], [42], [43], [44], [45]. Moreover, a potent and more sustained antiviral regimen should be considered for viral suppression to prevent the vicious cycle of inflammatory-cytokine activation in pH1N1 pneumonia especially among immunocompromised patients [8], [15]. Furthermore, our data suggested that future study on prognostication of severe influenza infection should consider the inclusion of cytokine/chemokine variables, because of their significant correlations with clinical progress and outcomes (e.g. a 10-fold increase in IL-6 is associated with >10 times higher risk for ICU admission)[8], [9], [15], [20], [21], [29], [31].

The strengths of our study include the comparisons of naturally-occurring, severe, influenza infections caused by different virus subtypes; patients were studied under the same clinical settings, using the same cytokine/chemokine assays. Samples were collected before antiviral treatment at baseline, and then serially during the hospital course [14], [15]. Corticosteroids for ‘viral pneumonitis’ and ‘ARDS’, which may confound the cytokine/chemokine responses was not given in our cohort [7], [19], [21]. Differences in patient demographic characteristics in pandemic and seasonal influenza should not affect our interpretation of data, since the host response patterns and the pathogenesis of severe forms of influenza disease were studied, which typically occur in the younger and older individuals respectively (in contrast to the study of the intrinsic properties of the viruses to induce cytokines). Further, the suppressed Th1/Th17 cytokine responses in severe pH1N1 pneumonia could not be easily explained by patient's younger age. Ideally, local (lung) production of cytokines should also be studied; however lower-respiratory secretion/tissue samples are difficult to obtain, and assay methods have not been standardized. Given that the lung is a highly vascular organ, it has been suggested that studying circulating cytokines may still provide a reasonably good approximation on their response patterns [15], [29]. We studied *ex vivo* cytokine expression of influenza patients' immune cells using WBA and PHA/LPS stimulation, instead of inactivated viruses that were available. This had avoided repetitive stimulation with a different virus strain, but we acknowledge the limitation that not all cytokine signaling pathways respond to such stimuli (e.g. IL–17A).

In conclusion, we found that in pH1N1 pneumonia the proinflammatory cytokines were hyperactivated, which correlated with severe symptoms and adverse outcomes. The adaptive immunity (Th-1/Th-17)-related cytokine responses were suppressed, in contrast to seasonal influenza. Cytokine/immune dysregulation likely plays an important role in the pathogenesis of severe, progressive influenza infection.
